# Sulphadoxine/pyrimethamine versus amodiaquine for treating uncomplicated childhood malaria in Gabon: A randomized trial to guide national policy

**DOI:** 10.1186/1475-2875-7-31

**Published:** 2008-02-12

**Authors:** Basile Nsimba, Vincent Guiyedi, Modeste Mabika-Mamfoumbi, Jean Romain Mourou-Mbina, Edgard Ngoungou, Marielle Bouyou-Akotet, Romaric Loembet, Rémy Durand, Jacques Le Bras, Maryvonne Kombila

**Affiliations:** 1 National Malaria Control Programme – Division for Disease Control, Ministry of Health, Brazzaville, Congo; 2 Department of Parasitology-Mycology and Tropical Diseases, Faculty of Medicine and Health Sciences, Libreville, Gabon; 3 Parasitology Laboratory, Avicenne Hospital, AP-HP, and EA 3406, Paris 13 University, Bobigny, France; 4 National Malaria Reference Centre, Bichat-Claude Bernard Hospital, Paris, France, and EA 209, University Paris Descartes, France

## Abstract

**Background:**

In Gabon, following the adoption of amodiaquine/artesunate combination (AQ/AS) as first-line treatment of malaria and of sulphadoxine/pyrimethamine (SP) for preventive intermittent treatment of pregnant women, a clinical trial of SP versus AQ was conducted in a sub-urban area. This is the first study carried out in Gabon following the WHO guidelines.

**Methods:**

A random comparison of the efficacy of AQ (10 mg/kg/day × 3 d) and a single dose of SP (25 mg/kg of sulphadoxine/1.25 mg/kg of pyrimethamine) was performed in children under five years of age, with uncomplicated falciparum malaria, using the 28-day WHO therapeutic efficacy test. In addition, molecular genotyping was performed to distinguish recrudescence from reinfection and to determine the frequency of the *dhps *K540E mutation, as a molecular marker to predict SP-treatment failure.

**Results:**

The day-28 PCR-adjusted treatment failures for SP and AQ were 11.6% (8/69; 95% IC: 5.5–22.1) and 28.2% (20/71; 95% CI: 17.7–38.7), respectively This indicated that SP was significantly superior to AQ (*P *= 0.019) in the treatment of uncomplicated childhood malaria and for preventing recurrent infections. Both treatments were safe and well-tolerated, with no serious adverse reactions recorded. The *dhps *K540E mutation was not found among the 76 parasite isolates tested.

**Conclusion:**

The level of AQ-resistance observed in the present study may compromise efficacy and duration of use of the AQ/AS combination, the new first-line malaria treatment. Gabonese policy-makers need to plan country-wide and close surveillance of AQ/AS efficacy to determine whether, and for how long, these new recommendations for the treatment of uncomplicated malaria remain valid.

## Background

Chloroquine (CQ) resistance is widespread in Gabon and this forced the health authorities to revise the national malaria treatment policy [1,2]. In July 2003, following a national consensus meeting, the Gabonese Ministry of Health responded to WHO recommendations by adopting the amodiaquine/artesunate (AQ/AS) combination as first-line treatment, the artemether/lumefantrine (A/L) combination as second-line treatment, sulphadoxine/pyrimethamine (SP) for preventing malaria in pregnant women; quinine remains the treatment of choice for severe malaria. Despite the considerable efforts of the Global Fund to help fighting malaria, the implementation of the WHO-recommended artemisinin-based combination treatments (ACTs) for controlling malaria encounters tremendous challenges in low-income countries, particularly in sub-Saharan Africa, because of the high cost of the drugs. Moreover, sound data supporting this new antimalarial-treatment policy is lacking in Gabon, and most in vivo studies to evaluate efficacy have been conducted with variable methodologies. Most previous studies were carried out in three provinces, namely Estuaire, Moyen-Ogooué and Haut-Ogooué, but none in Gabon's other six provinces. Consequently, studies carried out at the national level according to a standardized WHO protocol [3] were needed to determine the efficacy level of all antimalarial drugs to be used in the new national malaria treatment policy.

According to the data reported in the literature, *Plasmodium falciparum *CQ resistance was first reported in vitro in Gabon in 1983 [4] and confirmed by subsequent studies conducted throughout the country between 1984 and 1985 [5]. In 1987, CQ resistance was reported to have spread very fast, with an average reported rate of 42% [5]. Although this level of CQ resistance has remained stable since [6,7], there was a need to change this treatment policy. The situation was similar in neighbouring countries [8-10]. It has been reported that an increase of infant mortality was attributable to the escalating *P. falciparum *resistance to CQ [11]. In Gabon, CQ has been used for many years as first-line drug for treating uncomplicated malaria and for preventing malaria. AQ and SP are the only cheap and widely available antimalarials and they were used together as second-line drugs before the change in drug policy. Regarding AQ efficacy, Gabon is an unusal case amongst Central African countries, because of regional differences. All studies carried out in Libreville (Estuaire province) demonstrated a good efficacy of AQ, with resistance rates below 15% [1,2,12], while those conducted in Lambarané (Haut-Ogooué province) and in Bakumba (Moyen-Ogooué province) reported unacceptable resistance rates, higher than 25% [13,14]. A better understanding of the factors responsible for regional differences in AQ efficacy would be helpful to guide anew malaria treatment policy. The only previous studies assessing efficacy of SP were conducted in Franceville (Haut-Ogooué province) reporting a good efficacy of SP with clinical resistance rates below 10% [15]. A clinical trial testing the efficacy of AS monotherapy conducted in Lambarané showed an excellent clinical efficacy with a PCR-corrected 28-day cure rate of 90% [16].

If the strategy of combining two or more antimalarial drugs is widely recommended as a means for improving treatment efficacy and delaying the development of resistance, a knowledge of the resistance level against individual components of the combination is essential. Policy-makers in Gabon urgently need to know whether the newly-adopted malaria treatment strategy is a viable option in terms of therapeutic efficacy. They also need to learn how best to use information from clinical trials for optimizing the policy or considering when to make a further change in treatment policy.

The objectives of this study were to determine the efficacy level of AQ and SP using the standard WHO 28-day therapeutic efficacy test, with PCR-genotyping to distinguish recrudescences from reinfections due to therapeutic failures. Additionally, the prevalence of the *dhps *K540E mutation, previously defined as a predicting tool of SP-resistance [17], was also assessed. This is the first such study conducted in Gabon using standard WHO protocols.

## Patients and Methods

### Study area

Gabon is a Central African country bordering Equatorial Guinea and Cameroon to the north, and the Republic of Congo to the east. It has a 800 km coastline, providing access to the Atlantic Ocean to the west. It is situated on the equator and, hence, has a hot and humid climate, with precipitation nine months out of twelve. A dense forest covers 85% of its territory. Officially, Gabon's population is approximately 1.5 million, with 80% of the population living in towns. There are eight main ethnic groups, which are spread out across the country, but more than forty different ethnic groups altogether. On an administrative level, Gabon is subdivided into nine provinces, namely: Estuaire; Haut-Ogooué; Moyen Ogooué; Ngounié; Nyanga; Ogooué-Ivindo; Ogooué-Lolo; Ogooué-Maritime; and Woleu-Ntem. The rainy season is from October to May, and the dry season is from June to September. Malaria transmission is perennial with seasonal fluctuations.

### Study site

This study was carried out between March-July 2005 in Oyem, a town of the Woleu-Ntem province of 35,000 inhabitants, near the northern borders with Cameroon and Equatorial Guinea, and 411 km away from Libreville (the political and administrative capital) to the south-west. Malaria is hyperendemic and transmission is perennial. In this forest region, where *Anopheles gambiae s.s *is considered to be the most important vector of malaria, the *Plasmodium *index in children under 15 years of age is ranging from 65% to 73%, indicating an intense malaria transmission [18].

### Patient recruitment

The study was conducted in fchildren with fever, below five years of age, attending the Out-patient Department of Oyem Hospital. Using the 28-day WHO therapeutic test for intense transmission areas of malaria [3], patients were enrolled in the study if they satisfied the following inclusion criteria: age between 6–59 months, mono-infection with *P. falciparum*, parasitaemia ≥ 2,000 asexual parasites per μL of blood, free from severe malnutrition, absence of general danger signs or severe malaria, an axillary temperature of 37.5°C or above, absence of febrile conditions caused by diseases other than malaria, ability to come for the stipulated follow-up visits and easy access to the health facility, informed consent of parent/guardian, absence of history of hypersensitivity reactions to sulphonamides, and at least 5 g haemoglobin (Hb)/dL. Neither a history of previous antimalarial drug use, nor the presence of antimalarial drugs in the urine was an exclusion criterion in the WHO standard protocol [3,19]. Before enrolment in the study, a medical history of each patient was obtained from their accompanying parent or guardian and the child was clinically examined by a physician. Body weight and axillary or ear temperature were recorded; thick and thin smears were Giemsa-stained (5% Giemsa R for 20 min.) for parasite identification and quantification. Parasitaemia (parasites/μL) was measured by counting the number of asexual parasites against 200 leucocytes in the Giemsa-stained thick blood smears, based on a mean count of 8,000 leucocytes per μL of blood. A slide was declared negative only after microscopic fields corresponding to at least 500 leucocytes had been checked. Two experienced technicians performed the microscopy independently, each time comparing their results. The principal investigator closely supervised the study team to ensure consistency and accuracy of the data. Some slides randomly chosen were re-read in the laboratory in Paris.

Sample size estimations for the therapeutic test were performed by assuming that the treatment failures rate would be significantly less than 15% for SP and AQ, referring to previous clinical trials outcomes. Thus, according to WHO recommendations, a minimum of 50 patients was required for each therapeutic group [3].

### Patient treatment and follow-up

After obtaining informed consent from parents or guardians on day 0, the enrolled children were randomly allotted to one of two treatment groups, to receive either AQ base (Camoquin^® ^tablets, Pfizer, Dakar, Senegal) 30 mg/kg body weight over three days (i.e. 10 mg/kg daily), or SP (Maloxine^® ^tablets, Exphar, Belgium) as a single dose of 25 mg/kg of sulphadoxine/1.25 mg/kg of pyrimethamine.

An antipyretic (paracetamol, 15 mg/kg, every 8 h for 24 h) was systematically given on day 0 and if needed on day 1 and 2. All tablets were administered orally by a nurse in the presence of the physician. For 30 min following drug administration, patients were observed for vomiting and other side-effects. The same dose was re-administered if vomiting occurred. On days 1 and 2, symptoms, other medications, temperature, and physical examination were recorded, but microscopy was not performed unless one or more of danger signs were present. The same clinical observation was repeated and parasitological examination was conducted on days 3, 7, 14, 21 and 28. On day 0, when the patient presented a fever without parasitaemia in the absence of another pathology the child was seen on the next day for intensive follow-up and microscopic diagnosis of malaria. All treatment failures were treated with quinine tablets (8 mg/kg base three times daily for seven days).

### Classification of treatment outcomes

The responses to drug treatment were classified according to the WHO protocol [3], as an adequate clinical and parasitological response (ACPR), early treatment failure (ETF), and late treatment failure (LTF), including late clinical failure (LCF) and late parasitological failure (LPF).

### Molecular genotyping of parasite isolates

Finger-prick blood samples blotted on Isocode^® ^filter papers (Schleicher and Schuell, Ecquevilly, France) were dried and stored at room temperature in small separated and sealed plastic bags, prior to genotyping analysis at the National Malaria Reference Centre, Bichat-Claude Bernard Hospital, Paris, France. DNA was extracted with chelex-100 resin as previously reported [20]. Merozoite surface protein-2 (msp-2), which has been shown to be sufficiently discriminating in African parasite populations [21], was amplified by PCR for paired pre-treatment and failure samples to distinguish recrudescences from reinfections, as previously described [22]. Genotyping results PCR-adjusted were classified into three categories: 1) true recrudescences, 2) reinfections, and 3) mixed results (recrudescence and reinfection). Treatment outcomes with PCR correction were based on the number of recrudescences and reinfections, as indicated elsewhere [23]. The cases of mixed results were considered as recrudescences, and unconclusive results (PCR-DNA amplification failure) were excluded from the analysis. In addition, PCR-genotyping followed by sequencing method, as previously reported [17], was performed for assessing the prevalence of *dhps *K540E mutation.

### Ethical considerations

The local health and institutional authorities approved the research protocol (Gabonese Ministry of Public Health). Verbal and written informed consent for participation were obtained from parents or guardians, after thorough information on the study was provided in the local language.

### Statistical analysis

Data were analysed using version 2000 of the Epi-info software (Centers for Disease Control and Prevention, Atlanta, GA) and Graphpad Instat software (Graph Pad software, 10855 Sorrento Valley Road #203, San Diego CA92121 USA). Proportions were compared by performing Fisher's Exact test. The therapeutic response at day-28 of follow-up with its corresponding 95% confidence intervals was calculated using an intention-to-treat analysis, which included all patients who fulfilled enrolment criteria. Standard deviation (SD) was generally indicated for means and *P*-values of < 0,05 were calculated to demonstrate differences statistically.

## Results

Of the 632 febrile children with suspected malaria who were screened, three hundred-two (47.8%; 302/632) had confirmed malaria, of whom 154 met the inclusion criteria. Three hundred forty-four (54.4%; 344/632) were male; their mean age was 32.2 months.

**Figure 1:**
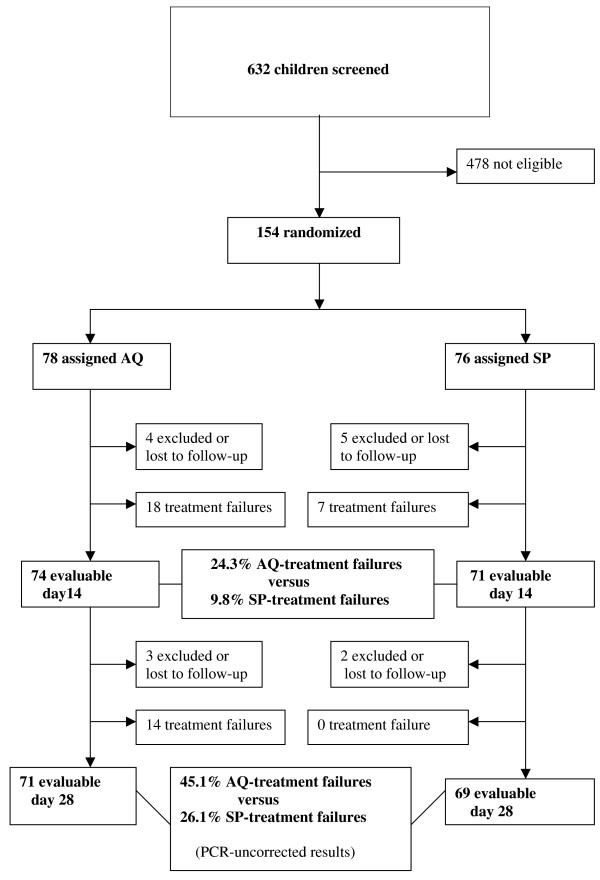
Trial Profile. AQ = amodiaquine; SP = sulphadoxine/pyrimethamine.

Of the 154 enrolled children, 78 were randomly assigned to AQ and 76 to SP. No difference was observed between the two therapeutic groups in terms of gender, mean age, mean body weight, presence of documented fever (axillary temperature ≥37.5°C) and parasitaemia. Overall, fourteen patients were excluded or lost to follow up (seven in AQ group and seven in SP group) during the follow-up period because of failure to follow the protocol (antimalarial treatment administered by themselves or a third party) or failure to come for follow-up on the scheduled days (generally because of travel for several days outside the city). Details of the patient follow-up are reported in Figure 1 (trial profile). No severe adverse drug reactions were observed during the follow-up of the patients in any therapeutic group. The day-28 PCR-adjusted failure rates were significantly higher in the AQ (28.2% 20/71; 95% CI: 17.7–38.7) group than in the SP group (11.6% 8/69; 95% CI: 5.5–22.1), (*P *= 0.0195). Of the 32 late treatment failures recorded in AQ group, detailed PCR-genotyping results were as following: 13 recrudescences, six reinfections, seven mixed results (recrudescence + reinfection) and six unconclusive results (PCR-amplification failure). Regarding SP group, detailed PCR-genotyping results among 14 late treatment failures were as following: eight true recrudescences and six reinfections. SP was superior to AQ for preventing recurrent infections (Table 1). The *dhps *K540E mutation was not found among the 76 analysed blood samples from patients allocated to the SP group.

**Table 1:** Efficacy at day-28 of sulphadoxine/pyrimethamine (SP) versus amodiaquine (AQ)

	SP			AQ			
		
	%	n	95% CI	%	n	95% CI	*P *value
ACPR	73.9	51	61.7–83.4	54.9	39	42.7–66.6	0.03
ETF	2.9	2	0.5–11.0	0	0	0.0–6.4	0.46
LCF	1.4	1	0.2–7.8	5.6	4	1.8–14.5	0.38
LPF	21.7	15	13.1–33.6	39.4	28	28.3–51.8	0.04
**Failure rate**							
Before PCR-correction	26.1	18/69	15.7–36.5	45.1	32/71	33.4–57.3	0.02
After PCR-correction	11.6	8/69	5.5–22.1	28.2	20/71	17.7–38.7	0.019

ACPR = adequate clinical and parasitological response; ETF = early treatment failure; LTF = late treatment failure; LCF = late clinical failure; LPF = late parasitological failure

## Discussion

The results of this study show, both clinically and parasitologically, that SP is significantly superior to AQ in terms of efficacy, with day-28 PCR-corrected failure rates of 11.6% and 28.2% respectively. SP also was more effective than AQ for preventing recurrent infections. Both regimens were safe and well-tolerated, with no serious adverse reaction recorded during the course of the study. Although protocols of previous studies were not in conformity with the standard WHO protocol, our data concerning SP efficacy are consistent with those reported in other parts of the country, such as in Bakoumba (Haut-Ogooué province) indicating a treatment failure rate of 14% at day 28 without PCR-adjustment in children under 10 years [14]. A good efficacy of SP was also observed in neighbouring Congo, Cameroon and Equatorial Guinea [9,10,24]. The absence of the *dhps *K540E mutation observed in the present study was already described in Gabon [25] and in neighbouring countries [17,25,26]. There is now a clear evidence that the principal role attributed to the *dhps *K540E mutation as a molecular marker predicting clinical SP-treatment failures is not true either in the existing literature or in the present study where 11.6% of SP resistance was found without a single instance of *dhps *K540E mutation. The AQ-resistance level reported in the study is too high, according to WHO guidelines (AQ-treatment failures >25%). The present results regarding AQ efficacy are consistent with other studies from Lambarané (Moyen-Ogooué province) and Bakumba (Haut-Ogooué province), reporting worse AQ efficacy with PCR-uncorrected resistance rates ranging from 34.7% to 47% on day 28 [13,14]. In contrast, the results from Libreville (Estuaire Province) have reported low AQ-failure rates ranging from 0% to 13% on day 14 [1,2,12]. Though these data are lacking PCR-adjustment, it seems that AQ efficacy is creating geographic differences in Gabon. Further AQ efficacy studies carefully carried out according to standard WHO protocol in other parts of the country, including settings reporting high AQ-failure rates, are urgently needed. Geographic differences of AQ efficacy cannot be directly explained by differences in malaria transmission levels between different regions of the country, because it was reported that transmission intensity does not directly affect the evolution of drug-resistance [27]. However, the relative importance of immunity in determining the response to therapy has been demonstrated in other studies, which had shown that age, a surrogate marker of acquired immunity, is associated with the ability to clear parasites with resistant genotypes [28]. It has been reported that cytochrome P450 CYP2C8 is involved in the metabolism of AQ, and additionally some information is currently available concerning its variation among ethnic groups as in Zanzibar and in Ghana [29,30]; this also needs to be investigated in Gabon in order to understand these inter-provincial differences of AQ efficacy.

In conclusion, geographical differences of AQ efficacy discussed in this study are a major concern in Gabon now that the AQ/AS combination has been selected as the new malaria first-line treatment policy. Thus, further investigations carefully conducted in the respect of good clinical practices are necessary to ascertain AQ-resistance at the national level.

In a country where ACTs are implemented, classical antimalarial drugs, including AQ and SP, must be monitored by recruiting persons with asymptomatic *P. falciparum *infection. The results of this longitudinal prospective study will provide an essential baseline of the parasitological efficacy of those monotherapies and also to be able to understand, at a later stage, the natural history of the evolution of resistance to ACTs.

## Authors' contributions

BN, VG, RD, JLB and MK designed the study and contributed to the discussion. MMM, JRMM, EN, MBA and RL participated to the clinical study. JRMM, BN, RD processed samples and analysed the data. BN wrote the first draft of the manuscript, then VG, RD, JLB and MK critically reviewed the manuscript. All authors read and approved the final manuscript.

## Conflict of interest

The author(s) declare that they have no competing interests.
